# Unique Case of Spontaneous Left Main Coronary Dissection in Second Trimester of Pregnancy Successfully Treated with Percutaneous Coronary Intervention: A Happy Ending

**DOI:** 10.3390/jcdd9010009

**Published:** 2021-12-31

**Authors:** Francesca Mantovani, Alessandro Navazio, Giovanni Tortorella, Vincenzo Guiducci

**Affiliations:** 1 Cardiology, Azienda Unità Sanitaria Locale–IRCCS di Reggio Emilia, Viale Risorgimento 80, 42123 Reggio Emilia, Italy; alessandro.navazio@ausl.re.it (A.N.); vincenzo.guiducci@ausl.re.it (V.G.); 2 Azienda AUSL Parma-Presidio Ospedaliero Di Fidenza Vaio, Via Don Enrico Tincati 5, 43036 Parma, Italy; gtortorella@ausl.pr.it

**Keywords:** spontaneous coronary artery disease, pregnancy, percutaneous coronary intervention

## Abstract

Among pregnant women, SCAD is the most frequent etiology of non-atherosclerotic acute coronary syndrome. SCAD related to pregnancy is more frequent within the first month (especially first week) of puerperium or last trimester, or is otherwise anecdotal. The concomitance of SCAD and pregnancy poses many issues regarding diagnosis and treatment in respect to maternal and fetal safety and requires tailored intervention with close interaction between clinical cardiologists, interventional cardiologists, cardiothoracic surgeons, and obstetricians. We report the case of a patient, pregnant in the second trimester with a life-threatening SCAD, successfully treated with percutaneous coronary intervention with excellent outcome for mother and baby.

## 1. Introduction

Spontaneous coronary artery dissection (SCAD) represents the most common etiology of pregnancy-associated myocardial infarction and is a frequent cause of acute coronary syndrome in young patients without atherosclerotic coronary artery disease [1,2].

The majority of cases occur late in the third trimester (with a mean of 32 weeks of gestation) or during the first month after delivery [3,4] with the highest frequency of SCAD in the first postpartum week [5] and it represents a dramatic and potentially fatal event, which remains poorly characterized and clinically under-recognized. Moreover, the concomitance of SCAD and pregnancy poses many issues regarding diagnosis and treatment in respect to maternal and fetal safety and requires carefully tailored intervention with close interaction between clinical cardiologists, interventional cardiologists, cardiothoracic surgeons, and obstetricians [6,7]. 

To our knowledge there are only two cases in the literature so far reporting a SCAD in the second pregnancy trimester [8,9], and in both cases, a fetal loss was reported. We report a unique case of SCAD in the second trimester of pregnancy successfully treated with percutaneous coronary intervention with good long-term outcomes for both mother and baby.

## 2. Case Report

A 33-year-old primigravid woman at 19-weeks’ gestation presented to the emergency room with ongoing chest pain. The patient’s medical history was unremarkable, with no traditional coronary artery disease risk factors. There was no family history of Marfan syndrome, sarcoidosis, connective tissue disorders, or vasculitis. She was not on medications and she reported no allergies. Her social history was unremarkable for alcohol, tobacco, or illicit drug use. On physical examination, she was afebrile, with a blood pressure of 100/60 mmHg, heart rate of 55 bpm, and with oxygen saturation of 99% in ambient air. Cardiac examination revealed normal heart sounds without murmurs, rubs, or gallops. Lungs were clear to auscultation bilaterally. The chest x-ray was unremarkable. The electrocardiogram (ECG) during symptoms revealed normal sinus rhythm with marked and diffuse anterolateral ischemic changes with ST-depression, with regression of these alterations when chest pain disappeared.

First, troponin-I was mildly positive at 0.019 ng/dL mild, whereas the rest of blood tests were unremarkable including the normal blood count, D-dimer, and serum electrolytes. The patient was admitted to the Coronary Care Unit. Transthoracic echocardiography showed a normal left ventricular function with no segmental wall motion defects. In accord with obstetricians, low molecular weight heparin (4000 IU, twice daily) and low-dose oral aspirin therapy were initiated promptly. Based upon presentation and test findings, a SCAD was suspected. The day after the patient was clinically and hemodynamically stable. The indication for coronary angiography was discussed in a multidisciplinary team, and taking into account the patient’s will, a watchful strategy was chosen.

**Figure 1:**
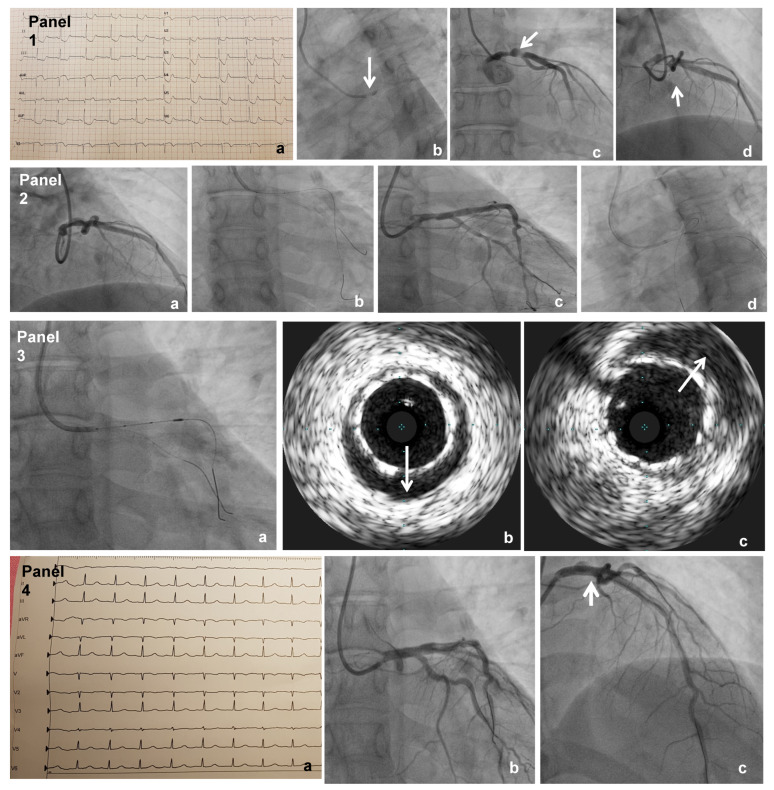
**Panel 1**(a): ECG before coronary angiography showing diffuse anterior ischemic changes; (b): basal coronary angiogram with indirect signs of left main intramural hematoma with low contrast media injection in the aortic root; (c): left main sub-occlusion due to compression of intramural hematoma; (d): dissection with residual true lumen involving ostium of the anterior descending artery (white arrows). **Panel 2**. Step by step procedure. (a): double wiring with balance middle weight into the circumflex and anterior descending artery; (b): stenting with Xience Pro 4 × 23 mm into LM-LAD system; (c): rewiring with Pilot 50 into CX and post-expansion with 4.5 × 15 mm non-compliant balloon Accuforce; (d): lateral kissing with 2 × 10 mm Euphora. **Panel 3**. Intra-procedural intravascular ultrasound assessment (Volcano Corporation) (a): catheter manipulation into LAD-LM before and after balloon dilatations; (b): residual malposition after direct stenting; (c): good stent strut apposition and persistence of intramural hematoma after high pressure non-compliant balloon post-expansion. (white arrows) **Panel 4**(a) ECG with regression of ischeamic changes after procedures, (b) and (c): Final coronary angiograms showing the restoration of normal flow and persistence of residual intramural hematoma (white arrow in (c)).

In the following days the patient was persistently asymptomatic and stable and was able to walk with no symptom occurrence. After 8 days, the patient suddenly experienced a new onset of chest pain and shortly after became hypotensive. ECG showed diffuse anterior ischemic changes; (Figure 1 Panel 1(a)). Bedside echocardiography documented hypokinesia of mid-septal and apical segments of the left ventricle with an ejection fraction of 45%. After a rapid, extended “heart team” (clinical cardiologists, interventionalists, cardiac surgeon, and obstetricians) discussion, a coronary angiography was urgently performed. Shielded protection was applied to the maternal pelvis in order to protect the fetus from X-ray exposure. The coronary angiography was performed from a radial artery access and showed a sub-occlusive left main (LM) dissection extending down to the left anterior descending artery (LAD) complicated by intramural hematoma (Figure 1 panel 1(b–d);). The left circumflex (CX) and dominant right coronary arteries were normal. According to clinical conditions, a decision was taken to treat immediately the LM dissected bifurcation with a provisional stenting technique. PCI was preferred over CABG in favor of suitable bifurcation anatomy without a diseased side branch and in order to minimize the risk of fetal damage/complications deriving from CABG surgery.

The intervention was performed using a 6 French guiding catheter (EBU 3.5 Medtronic). The procedure consisted of double wiring into LAD/CX (0.014 Balance Middle Weight) passing through the true lumen of the dissection, then stenting with a 4 × 23 mm everolimus-eluting stent (Xience Pro Abbott Vascular) into an LM-LAD system, before finally rewiring (0.014 Pilot 50) into CX and stent post-expansion with a 4.5 × 15 mm non-compliant balloon (Accuforce Terumo) and lateral kissing with a 2 × 10 mm semi-compliant balloon (Euphora Medtronic) (Figure 1 panel 2(a–d)). Intra-procedural intravascular ultrasound (Volcano Corporation) performed before and after the high-pressure non-compliant balloon showed stent struts well apposed, no residual dissection, and the persistence of mild intramural hematoma (Figure 1 panel 3(a–c)). The final angiograms confirmed stent patency and restoration of normal flow in multiple views (Figure 1 panel 4(b,c);). The invasive procedure ended with a total amount of media contrast assigned < 150 milliliters and an insignificant fetal X-ray exposure measured (0.02 millisievert). ECG normalized (Figure 1 panel 4(a)). The patient tolerated the procedure well, recovered uneventfully, and was discharged on prasugrel (10 milligrams once a day) and low-dose aspirin (100 milligrams once a day). The good fetal status was confirmed by a post-procedural echography. The patient’s recovery and the subsequent pregnancy was otherwise without incident. 

An elective cesarean section under spinal anesthesia was the method chosen for delivery in order to reduce maternal stress. Considering the planned intervention with intermediate risk of bleeding and the patient at high thrombotic risk, prasugrel was discontinued 7 days before the procedure and bridged with enoxaparin, while low-dose aspirin was continued. Delivery was uncomplicated, with the birth of a healthy baby boy.

No maternal complications were recorded and prasugrel was resumed 3 days later, with an agreement not to breastfeed. Five days later, our patient returned home with her son. The patient was counselled against subsequent pregnancies.

At 6-month and 1-year follow-ups, the patient underwent CT-coronary angiography showing patent stents. The patient and her baby are well to date.

## 3. Discussion

The present case is unique in the literature so far for various reasons and therefore may offer teaching points.

First, SCAD in the second trimester of pregnancy is anecdotal [6,8,9] and has occurred only once during an uncomplicated pregnancy [9]. In a recent series of pregnancy-related SCADs described by Tweet et al. [5] only 4/54 women were pregnant at the time of SCAD and only one < 28 weeks, and in 13 patients with SCAD related to pregnancy reported by Cade et al. only one was pregnant (in the 37th week) [10]. This underscores the need for awareness of this condition in cardiology and obstetrics as SCAD may present during all stages of pregnancy.

Secondly, an initial watchful waiting was decided upon as the young maternal age and the absence of coronary risk factors for atherosclerotic disease made SCAD the most plausible diagnosis and contemporary observational data indicate that conservative management of hemodynamically stable patients without ongoing signs of ischemia generally results in spontaneous healing of dissections [11,12] and favorable outcomes [11,13]. However, as previously described [1,12,14], early complications of recurrent acute coronary syndrome following SCAD may occur, most commonly over the first five days/first week and our patient required urgent revascularization. Even if coronary angiography with X-ray exposure and the use of a contrast agent is considered to pose high risk of permanent damage to the fetus, it may be performed with relatively low fetal radiation exposure, and vascular access via the upper extremity avoids direct fetal irradiation during catheter passage. Typical expected estimated fetal doses associated with maternal cardiovascular X-ray imaging are typically described as being as low as < 0.07 millisievert [7], and it has been shown to have been even lower in our case; therefore, this should not be a barrier when these procedures are clinically indicated.
